# Mucosal vaccines for SARS-CoV-2: scientific gaps and opportunities—workshop report

**DOI:** 10.1038/s41541-023-00654-6

**Published:** 2023-04-12

**Authors:** Jane M. Knisely, Lucas E. Buyon, Rebecca Mandt, Rebecca Farkas, Shobana Balasingam, Karin Bok, Ursula J. Buchholz, M. Patricia D’Souza, Jennifer L. Gordon, Deborah F. L. King, Tung T. Le, Wolfgang W. Leitner, Robert A. Seder, Alkis Togias, Stig Tollefsen, David W. Vaughn, Daniel N. Wolfe, Kimberly L. Taylor, Anthony S. Fauci

**Affiliations:** 1 Office of the Director, National Institute of Allergy and Infectious Diseases, National Institutes of Health, Bethesda, MD 20892 USA; 2 Division of Microbiology and Infectious Diseases, National Institute of Allergy and Infectious Diseases, National Institutes of Health, Bethesda, MD USA; 3 Office of Scientific Management and Operations, National Institute of Allergy and Infectious Diseases, National Institutes of Health, Bethesda, MD 20892 USA; 4 Coalition for Epidemic Preparedness Innovations, Skøyen Atrium, Askekroken 11, 0277 Oslo, Norway; 5 Infectious Disease, Prevention, Wellcome Trust, UK, London, NW1 2BE UK; 6 Vaccine Research Center, National Institute of Allergy and Infectious Diseases, National Institutes of Health, Bethesda, MD USA; 7 RNA Viruses Section, Laboratory of Infectious Diseases, National Institute of Allergy and Infectious Diseases, National Institutes of Health, Bethesda, MD 20892 US; 8 Division of AIDS, National Institute of Allergy and Infectious Diseases, National Institutes of Health, Rockville, MD USA; 9 Division of Allergy, Immunology, and Transplantation, National Institute of Allergy and Infectious Diseases, Rockville, MD USA; 10 Bill & Melinda Gates Foundation, Seattle, WA USA; 11 Administration for Strategic Preparedness and Response, Biomedical Advanced Research and Development Authority, 200 C Street SW, Washington, DC 20024 USA

**Keywords:** Vaccines, SARS-CoV-2

## Abstract

On November 7th and 8th, 2022, The National Institute of Allergy and Infectious Diseases (NIAID), part of the National Institutes of Health (NIH), The Coalition for Epidemic Preparedness Innovation (CEPI), The Bill & Melinda Gates Foundation (BMGF), The Biomedical Advanced Research and Development Authority (BARDA), and the Wellcome Trust hosted a virtual workshop entitled “Mucosal Vaccines for SARS-CoV-2: Scientific Gaps and Opportunities.” During the workshop, researchers and vaccine developers from around the world discussed the potential of mucosal vaccines to block SARS-CoV-2 transmission and reviewed the status of SARS-CoV-2 mucosal vaccine research. Here, we summarize key challenges and opportunities in basic, translational, and clinical research that were highlighted during the meeting. We also provide recommendations to advance the field and accelerate the development of mucosal vaccines for SARS-CoV-2.

## Why consider mucosal vaccines for SARS-CoV-2?

Currently available COVID-19 vaccines are highly effective at preventing severe disease. However, they may not adequately prevent infection or transmission of virus, especially over time, as evidenced by the continued spread of SARS-CoV-2 in highly vaccinated populations, especially with the emergence of new variants^1,2^. Ongoing community transmission and virus replication allows for the evolution and emergence of new immune-evasive variants that limit the effectiveness of current COVID-19 vaccines in preventing infection and symptomatic disease^2–5^. A priority goal for next-generation COVID-19 vaccines is to reduce infection with and transmission of SARS-CoV-2 via the respiratory route, while maintaining or enhancing protection against symptomatic and severe disease. Utilization of mucosal vaccination to elicit robust mucosal immunity in the respiratory tract is a logical approach to achieving these goals^1,6^.

Vaccines that induce a durable mucosal immune response localized in the respiratory tract have the potential to prevent virus infection, replication, and shedding and therefore, transmission^1^. Even in the event of infection, mucosal immunity to SARS-CoV-2 at the primary site of viral shedding may prevent forward transmission^1^, and, in the context of adequate vaccine uptake, lower the incidence of COVID-19 infection, thereby slowing the emergence of variants, and reducing the probability of future disease surges and attendant acute and post-acute morbidity. Moreover, knowledge gained from the development of effective mucosal SARS-CoV-2 vaccines could be applied to other respiratory pathogens, both known and yet- to- emerge^1^. Mucosal vaccines also offer the advantage of needle-free administration, which could lower barriers to access for some populations and improve vaccine uptake^7^.

On November 7th and 8th 2022, The National Institute of Allergy and Infectious Diseases (NIAID), a component of the National Institutes of Health (NIH), The Coalition for Epidemic Preparedness Innovation (CEPI), The Bill & Melinda Gates Foundation (BMGF), The Biomedical Advanced Research and Development Authority (BARDA), and the Wellcome Trust invited vaccine researchers and developers from around the world to: (1) explore the potential of mucosal vaccines to block infection with and transmission of SARS-CoV-2; (2) define challenges and opportunities in SARS-CoV-2 mucosal vaccine research; and (3) discuss key characteristics of mucosal vaccines for COVID-19. Presentations and discussions took place over eight sessions (materials can be found at https://web.cvent.com/event/c7bc69cc-9159-48a3-875e-beef880b8547/websitePage:3f0ae85c-4848-4d57-bfdd-5a24bedbbde2?locale=en-US), spanning basic research questions to regulatory and policy considerations. In this report, we highlight high-level takeaways from the meeting and discuss key research gaps and opportunities in basic, translational, and clinical research to advance the development of mucosal vaccines for SARS-CoV-2.

## Landscape of mucosal SARS-CoV-2 vaccines

As of December 2022, five mucosal vaccines for SARS-CoV-2 have been authorized for use or registered to be reviewed by a regulatory agency, though none have been authorized in the United States or Europe or achieved Emergency Use Listing by the WHO. An additional 16 are in clinical development (Table 1), and according to a review of publicly available data at least 44 are in preclinical development at various levels of different animal model testing. These vaccines utilize a variety of vaccine platforms (protein-based, viral-vectored, live-attenuated virus, DNA, RNA, and inactivated virus) and delivery modalities (nasal and oral dropper, sprayers (aerosolized), inhaler, nebulized delivery, and oral tablet delivery). The preclinical and clinical development pipeline is currently dominated by protein-based technologies and viral vectors.

**Table 1:** List of SARS-CoV-2 mucosal vaccine candidates in use or in clinical development.

Lead developer	Country	Platform	Route of delivery	Development stage
**Bharat Biotech**	India	Viral vector	Dropper	Registration/Introduction^63,64^
**CanSinoBIO**	China	Viral vector	Nebulizer	Registration/Introduction^65,66^
Gamaeleya	Russia	Viral vector	Sprayer/inhaler	Registration/Introduction^67,68^
Razi Institute	Iran	Protein-based	Sprayer/inhaler	Registration/Introduction^69^
Beijing Wantai	China	Live attenuated	Sprayer/inhaler	Registration/Introduction^70^
**Codagenix/Serum Institute of India**	United States/India	Live attenuated	Sprayer/inhaler	Clinical- Phase III^71^
**Mount Sinai/CastleVax**	United States	Viral vector	Sprayer/inhaler	Clinical-Phase II^72^
**VaxArt**	United States	Viral vector	Oral/tablet	Clinical-Phase II^73^
Center for Genetic Engineering and Biotechnology	Cuba	Protein-based	Sprayer/inhaler	Clinical-Phase II^74^
CyanVac LLC	United States	Viral vector	Sprayer/inhaler	Clinical-Phase I^75^
**AstraZeneca/Oxford**	**United Kingdom**	**Viral vector**	**Sprayer/inhaler**	**Clinical-Phase I**^76^
Tetherex/Moat Biotechnology	Australia	Viral vector	Other/unknown	Clinical-Phase I^77^
iosBio/ImmunityBio	United States	Viral vector	Oral/tablet	Clinical-Phase I^78^
**ACM Biolabs**	Australia	Protein-based	Dropper	Clinical-Phase I^79,80^
BlueWillow	United States	Protein-based	Sprayer/inhaler	Clinical-Phase I^81^
VaxForm	United States	Protein-based	Oral/suspension	Clinical-Phase I^82^
Yisheng Bio	China	Protein-based	Nebulizer	Clinical-Phase I^83^
Oravax/Oramed	United States/Israel	Protein-based	Oral/tablet	Clinical-Phase I^84^
Meissa	United States	Live attenuated	Sprayer/inhaler	Clinical-Phase I^85^
Symvivo	Australia	Live attenuated	Oral/tablet	Clinical-Phase I^86^
Intravacc	The Netherlands	Protein-based	Other/unknown	Clinical-Phase I^87^

Companies in bold presented at the workshop.

Notably, there is limited data available from clinical efficacy trials to assess their impact on transmission, infection, or disease; however, several candidates appear to induce a mucosal immune response based on immune markers, such as secretory IgA (sIgA) and secretory IgG (sIgG) although the clinical significance of those markers is currently unclear.

## Challenges and opportunities

The workshop highlighted the state of the basic, translational, clinical, and regulatory science that provides possible avenues to the development and approval of safe and effective mucosal vaccines for SARS-CoV-2. In addition, each workshop session focused on key challenges that will need to be overcome for the successful development of these vaccines.

### Mucosal immunology and correlates of protection

A better understanding of mucosal immunity and the key components that contribute to protection at the mucosal level is needed to inform the selection of vaccine platforms and adjuvants, route of delivery, and mucosal immune markers that should be measured in clinical trials. The current established immune correlate of protection against symptomatic disease^8^ is neutralizing antibody titers measured from blood. Importantly there are numerous basic research questions related to correlates of durable protection against the more recent variants of concern and how this would inform optimal next-generation vaccine design which could include mucosal delivery as well as clinical evaluation. Such topics include a better understanding of which cells, whether stimulated by natural infection or vaccination, are most critical for short- and long-term immunity and the nature of interactions among immune cells in the upper and lower airways, and gut^1,9–11^.

Several presenters across workshop sessions noted there is uncertainty about which mucosal immune markers are clinically meaningful. Neutralizing antibodies, binding antibodies through the Fc, anamnestic antibody responses, and T cells may contribute to protection in the lungs^1,12^. Neutralizing antibodies and T cells may also play a role in protection in the upper airway^1,13^. However, the low frequency of T cells induced though parenteral vaccination in the nose may limit that protection^14^. Induction of sIgA and sIgG antibodies in the airway, as well as the presence of airway CD4 + and CD8 + T cells specific to the SARS-CoV-2 spike protein play a role in mediating mucosal immunity^15^. The role of resident memory B cells producing IgG antibodies in maintaining mucosal immunity to *Streptococcus pneumoniae*^16^ was also highlighted, though their role in SARS-CoV-2 mucosal immunity remains to be elucidated. It was noted that the serological threshold of circulating antibodies for protection against mucosal infection may be higher than that required to prevent severe clinical outcomes^16^. Beyond systemic immunity of circulating antibodies, and B and T cells in the blood, other speakers reported that tissue-resident memory B and T cells in bronchoalveolar lavage (BAL) fluid provide immunity in the lower respiratory mucosa and are part of the protection mechanism. Other presenters noted that vaccinated individuals had substantially fewer spike-specific BAL tissue-resident memory CD4 + T, CD8 + T, and RBD-specific memory B cells compared with a convalescent group who had prior SARS-CoV-2 infection^13^. These observations are an important addition to other studies that have reported superior mucosal (saliva) antibodies in those with prior COVID-19 compared with vaccinated individuals^10^ and the establishment of tissue-resident T cells for up to 6 months after infection^17^. Data from the SARS-CoV-2 controlled human infection model (CHIM) studies conducted at Imperial College London in unvaccinated, uninfected participants suggest that a combination of immune factors, including cross-reactive antibodies against seasonal coronaviruses, the presence and number of cross-reactive non-structural protein (NSP)-specific T cells, and rapid induction of the innate immune response are also associated with either a lack of detectable virus replication on mucosal surfaces or the presence of infection that is only transient in nature^18–20^. It was cautioned, however, that these responses do not necessarily constitute sterilizing immunity. Persistent neutralizing antibodies in the upper airway may be required to prevent infection^1^. Speakers also noted that IgA antibodies have short persistence^21^, which may limit the transmission-blocking effectiveness of mucosal vaccines, or require boosting to maintain transmission-blocking efficacy. Determining longevity of IgA antibodies induced from mucosally delivered SARS-CoV-2 vaccines, and their role in transmission blocking is a significant research need.

The lack of validated correlates of protection for respiratory mucosal protection has implications for clinical research, as well as regulatory and policy considerations. As discussed during the Regulatory and Policy Considerations workshop session, next-generation COVID-19 vaccines can be approved by comparing systemic neutralizing IgG levels to those induced by approved vaccines, a process known as “immunobridging,” if similar platforms are used^22^. However, because there are no validated correlates of protection for respiratory mucosal protection, if a vaccine has a unique mode of action that elicits an effective mucosal response but does not induce the same systemic immune markers as current vaccines, it will need to undergo large and expensive Phase 3 trials to show clinical efficacy^23^. Furthermore, to generate evidence to support a policy recommendation for preferred use as a transmission-blocking vaccine, developers may have to include an infection or transmission endpoint and/or conduct large Phase 4 studies.

Despite these challenges, the growing body of work on SARS-CoV-2 infection and vaccination in both animal models and humans is rapidly expanding our knowledge of the sites where a mucosal immune response is induced, elucidating components of this response that are important for protection. Development of CHIM, single-cell RNA-seq, antigen-specific T-cell proliferation assays, highly sensitive mucosal antibody assays, and others, can facilitate the association of mucosal immune markers with clinical outcomes^18,24^. These studies and techniques can help identify immune correlates of transmission prevention, and—subsequently—promising vaccine candidates. Furthermore, these developments will advance the field of mucosal immunology for both SARS-CoV-2 and other mucosal respiratory pathogens.

### Animal models

The basic and translational research sessions highlighted multiple studies in animal models which demonstrated that intranasal vaccination can induce local immune responses in the respiratory mucosa and protect animals from infection and/or disease. In some studies, protection persisted for several months^25–28^, supporting the possibility of inducing effective mucosal immunity to SARS-CoV-2 via vaccination. However, there are key differences between the immune systems of animals and humans, as well as physical differences in the sizes and shapes of the airways and lungs. Because of this, animal models of SARS-CoV-2 transmission are not necessarily predictive of human biology. For example, vaccine administered intranasally may reach the lower airways in animals, but not in humans. This was illustrated through data presented on the intranasal administration of the ChAdOx1 COVID-19 / AZD1222 vaccine, which was shown to reduce intranasal shedding in Syrian hamsters, and subsequently transmission between hamsters^29–31^. However, the same vaccine did not consistently induce a mucosal immune response in a phase I trial in humans^32^. This comparison illustrates the limitations of inferring vaccine response, transmission dynamics, and vaccine efficacy in humans from animal studies and highlights the need for more predictive preclinical models of SARS-CoV-2 transmission.

### Clinical study designs

As for any candidate vaccine, adequate clinical safety experience will need to be generated to assess the potential risks of various platforms associated with mucosal delivery. Prior work demonstrated a link between intranasal adjuvanted influenza vaccination and risk of developing Bell’s Palsy^33,34^, raising concerns around the potential for adverse events when using pro-inflammatory adjuvants with nasal vaccination. Careful adjuvant selection and identification (low/no inflammatory profile) and formulation (for better targeting nasal mucosa) are critical research gaps to be addressed in order to prevent potential safety concerns. Speakers also highlighted the challenge of designing clinical studies to measure the impact of a vaccine on transmission. While reduced transmission of SARS-CoV-2 to household contacts has been demonstrated for some parenterally administered vaccines^35^, none of the current mucosal vaccine products or candidates have demonstrated efficacy against infection or transmission in humans, nor has this been required for authorization. CHIM may play a role in studies to dissect the underlying mechanism of transmission and correlates of protection, but these designs face major challenges, including long timelines for the production of challenge strains and difficulty establishing infection in individuals with pre-existing immunity^18,36^. Assessing prevention of transmission in the context of a randomized controlled trial (RCT) is possible but operationally challenging and resource intensive^35^. The RCT design also depends on the endpoint of interest. Two trial designs to assess secondary transmission were discussed; a prospective cohort study, which identifies study participants (including contacts) at study enrollment, or a case-ascertained close contacts study, which identifies close contacts at the time of diagnosis of the index patient^37,38^. Both study designs have challenges. The prospective cohort design reduces the potential for bias but is resource intensive and the recruitment of contacts at time of study onset may be challenging. The ascertained close contacts study is more resource-efficient but prone to bias from delayed identification and testing of contacts. For all RCT designs, the heterogeneous baseline immunity and age of the study population can make the analysis complex. Current US-based platforms used to determine vaccine effectiveness post-deployment^39,40^ are not designed to be able to assess transmission. In addition, the current ambiguity of mucosal markers as meaningful predictors of mucosal protection in humans creates uncertainty in what specimens to collect and what immune parameters to measure during clinical studies. Presenters noted that there is no determined “best practice” for collecting clinical samples (nasal swabbing, PMBC collection, serology and immunology determined by nasal wick) and measuring infectiousness (area under the viral load (VL) curve, VL at detection of infection, peak VL, duration of VL above a threshold, burden of infection as determined by RT-PCR, and isolation of virus from culture). There are many different approaches, each with pros and cons^41–47^. Harmonization of RCT study designs and standardization of clinical protocols, endpoints, and assays that will allow for evaluation of, and comparison between, candidates are critical needs.

Despite these challenges, there are approaches to conduct clinical studies of mucosal SARS-CoV-2 vaccines, and potential paths forward to licensure. Regulatory bodies have approved new SARS-CoV-2 vaccines through immunobridging^22^. Multiple mucosal vaccine candidates have demonstrated induction of both systemic and mucosal immune markers in preliminary clinical trial results^48–51^, indicating that licensure on this basis is possible; however, prevention of transmission would still need to be tested in Phase 4 studies in order to recommend mucosal vaccine use over intramuscular vaccines. Additionally, while studies to evaluate the impact of vaccines on infection and/or transmission can be challenging, they nonetheless are possible. Future studies can draw on lessons learned from earlier work, such as the Coronavirus Protection Network (Co-VPN)’s transmission study conducted in university students (https://clinicaltrials.gov/ct2/show/NCT04811664). While the study was ultimately halted due to enrollment issues, there were important takeaways based on this experience about what kinds of study designs are feasible, and what operational challenges would need to be overcome to successfully evaluate transmission. For example, study designs involving frequent swabbing may only be feasible for short periods of time. Building strong relationships between sites and the study population will likely be necessary to recruit, retain, and enroll and retain participants and their contacts, and ensure adherence to study protocols.

### Device considerations

The different routes of mucosal administration being explored raise additional considerations around manufacturing, supply chain, safety, feasibility, acceptability, and cost-effectiveness that will factor into regulatory and policy decisions. For example, while oral delivery or simple devices such as the nasal sprayer used to administer FluMist could make vaccine deployment easier^52^, other devices that are more complex to use, such as nebulizers, could limit the ability to deploy a mucosal vaccine^9,53,54^. Additionally, any product that involves a device will require manufacturing and approval of the device as well as the vaccine itself. In general, oral delivery options (pills, tablets, liquids) are the most straightforward to manufacture and deploy, followed by intranasal (droppers, sprayers) and then intratracheal (nebulizers, inhalers). It is important for developers to recognize that changing devices midstream will necessitate repeating studies.

The mucosal vaccine candidates discussed during the workshop highlighted how developers are beginning to identify effective administration routes. Seven candidates representing a range of vaccine platforms and delivery routes (bolded in Table 1) were selected to share data during the Company Presentations session. The CanSinoBIO adenovirus-vectored, Convidecia Air^55^ vaccine, licensed in China, induced higher levels of bronchial IgA when administered via the inhaled compared to the intranasal route in primates^56^. Bharat Biotech’s vaccine, approved in India, and the Oxford-AstraZeneca vaccine both use an adenovirus-vectored-spike approach, and are administered intranasally. However, they utilize different adenovirus serotypes, spike sequence, formulations, and delivery devices (dropper vs. sprayer), all of which may contribute to the different mucosal immune responses observed in clinical trials^57–59^. Data from previously-published work on oral norovirus and influenza vaccines^60,61^, and clinical trial results from VaxArt’s two SARS-CoV-2 vaccine candidates^62^ suggested an oral vaccine can induce a mucosal immune response in the upper respiratory tract and protect against respiratory pathogens. At least five other companies are also pursuing tablet delivery candidates (Box 1). A key advantage of the oral administration route is ease of manufacture and administration of an oral tablet, which does not require a specialized delivery device, and which could potentially even be self-administered at home.

Box 1 Key recommendations for the development of mucosal vaccines for SARS-CoV-2 • Continue investment in SARS-CoV-2 mucosal vaccines research and development

• Connect early-stage researchers with advanced development partners and de-risk candidates

• Identify mucosal correlates of protection and develop standardized assays and sampling protocols

• Develop animal models that are predictive of mucosal responses and transmission effects in humans

• Develop and harmonize study protocols to assess impact on infection and transmission

• Further research on optimal vaccine platforms, adjuvants, and administration routes

• Encourage regulators to align on authorization pathways.

### Use cases

With the notable exception of children, next-generation vaccines will largely be used in the context of pre-existing immunity from vaccination, prior infection, or both. Several workshop presenters highlighted the advantage of using mucosal vaccines as boosters following intramuscular vaccination. Animal model studies demonstrated that intramuscular mRNA vaccination followed by intranasal administration of either a protein or adenovirus-based vaccine induced stronger mucosal immune responses compared to a second mRNA boost, along with comparable systemic immune responses. In animal model studies, the intramuscular prime, intranasal boost strategy led to increased breadth and durability of neutralizing antibody responses, as well as improved protection against disease and faster viral clearance^13,15^. Data presented from vaccine developers suggested that this advantage may hold up in clinical settings. In two clinical trials of next-generation mucosal vaccine candidates, successful immune boosting in individuals who had previously received an intramuscular SARS-CoV-2 vaccine was shown with reporting of increased immune responses against both wild-type SARS-CoV-2 and variants of concern^49,50^.

## Conclusions and recommendations to advance the field

While SARS-CoV-2 mucosal vaccines have yet to directly demonstrate clinical utility, panelists expressed optimism given there are mucosal vaccines at all stages of development, including several that have achieved country-level approvals (Table 1). Over the next few years, continued development of these candidates and others, and expanded evaluation of authorized vaccines will inform the potential of one or more of these approaches to not only protect individuals from disease but to dramatically reduce virus transmission. Throughout the workshop, panelists discussed what knowledge, tools, and other resources are most needed to advance the field and lower barriers to mucosal vaccine development, resulting in several overarching recommendations (Box 1). Participants agreed that the potential benefit of mucosal vaccines warrants further investment of resources, focus, and coordination. Dedicated efforts to develop mucosal vaccines that induce durable protection against infection at the respiratory mucosa and limit the transmission of SARS-CoV-2 could, accompanied by significant vaccine uptake, bring an end to the current COVID-19 pandemic, while generating knowledge that could be applied to vaccines for current challenges such as RSV, tuberculosis and influenza, as well as potential future pandemic threats.
